# Eplerenone prevents salt-induced vascular stiffness in Zucker diabetic fatty rats: a preliminary report

**DOI:** 10.1186/1475-2840-10-94

**Published:** 2011-10-18

**Authors:** Markus Resch, Peter Schmid, Kerstin Amann, Sabine Fredersdorf, Joachim Weil, Christian Schach, Christoph Birner, Daniel P Griese, Peter Kreuzer, Sabine Brunner, Andreas Luchner, Günter AJ Riegger, Dierk H Endemann

**Affiliations:** 1 Department of Internal Medicine II, Regensburg University Medical Center, Franz Josef Strauss Allee 11, 93053 Regensburg, Germany; 2 Department of Nephropathology, University Hospital Erlangen, Universitätsstr. 22, 91054 Erlangen, Germany; 3 Medical Clinic II - Cardiology, University Hospital Schleswig Holstein Campus Lübeck, Ratzeburger Alle 160, 23538 Lübeck, Germany; 4 Department of Cardiology, Herz- und Gefäßklinik GmbH Bad Neustadt an der Saale, Salzburger Leite 1, 97616 Bad Neustadt an der Saale, Germany

## Abstract

**Background:**

Aldosterone levels are elevated in a rat model of type 2 diabetes mellitus, the Zucker Diabetic fatty rat (ZDF). Moreover blood pressure in ZDF rats is salt-sensitive. The aim of this study was to examine the effect of the aldosterone antagonist eplerenone on structural and mechanical properties of resistance arteries of ZDF-rats on normal and high-salt diet.

**Methods:**

After the development of diabetes, ZDF animals were fed either a normal salt diet (0.28%) or a high-salt diet (5.5%) starting at an age of 15 weeks. ZDF rats on high-salt diet were randomly assigned to eplerenone (100 mg/kg per day, in food) (ZDF+S+E), hydralazine (25 mg/kg per day) (ZDF+S+H), or no treatment (ZDF+S). Rats on normal salt-diet were assigned to eplerenone (ZDF+E) or no treatment (ZDF). Normoglycemic Zucker lean rats were also divided into two groups receiving normal (ZL) or high-salt diet (ZL+S) serving as controls. Systolic blood pressure was measured by tail cuff method. The experiment was terminated at an age of 25 weeks. Mesenteric resistance arteries were studied on a pressurized myograph. Specifically, vascular hypertrophy (media-to-lumen ratio) and vascular stiffness (strain and stress) were analyzed. After pressurized fixation histological analysis of collagen and elastin content was performed.

**Results:**

Blood pressure was significantly higher in salt-loaded ZDF compared to ZDF. Eplerenone and hydralazine prevented this rise similarily, however, significance niveau was missed. Media-to-lumen ratio of mesenteric resistance arteries was significantly increased in ZDF+S when compared to ZDF and ZL. Both, eplerenone and hydralazine prevented salt-induced vascular hypertrophy. The strain curve of arteries of salt-loaded ZDF rats was significantly lower when compared to ZL and when compared to ZDF+S+E, but was not different compared to ZDF+S+H. Eplerenone, but not hydralazine shifted the strain-stress curve to the right indicating a vascular wall composition with less resistant components. This indicates increased vascular stiffness in salt-loaded ZDF rats, which could be prevented by eplerenone but not by hydralazine. Collagen content was increased in ZL and ZDF rats on high-salt diet. Eplerenone and hydralazine prevented the increase of collagen content. There was no difference in elastin content.

**Conclusion:**

Eplerenone and hydralazine prevented increased media-to-lumen ratio in salt-loaded ZDF-rats, indicating a regression of vascular hypertrophy, which is likely mediated by the blood pressure lowering-effect. Eplerenone has additionally the potential to prevent increased vascular stiffness in salt-loaded ZDF-rats. This suggests an effect of the specific aldosterone antagonist on adverse vascular wall remodelling.

## Background

The renin-angiotensin-aldosterone system (RAAS) plays a key role in the pathophysiology of hypertension and vascular complications of diabetes mellitus. The mineralocorticoid aldosterone is a well established target in the pharmacotherapy of congestive heart failure and hypertension [1,2]. Specific inhibition of the mineralocorticoid receptor on top of ACE inhibitor medication has recently been implicated as a new therapeutic strategy to improve the coronary circulation in diabetics. Aldosterone antagonism has also demonstrated potential to protect from certain cardiovascular and other end-organ damage in animal models. Spironolactone or the more specific antagonist eplerenone prevented left ventricular inflammation and fibrosis [3-6], renal inflammation and proteinuria [7,8], stiffening of the carotid artery [9-12], resistance artery remodelling, endothelial dysfunction, and activation of NADPH oxidase [13,14]. Eplerenone has been shown to reduce resistance artery stiffness in hypertensive patients [15]. In diabetes mellitus aldosterone antagonism effectively reduces renal injury irrespective of the species or specific cause of diabetes [16]. We previously reported a state of hyporeninemic hyperaldosteronism in a model of type II diabetes mellitus, the Zucker Diabetic Fatty ZDF-rat [17]. However, it is unclear if specific aldosterone antagonism is also able to prevent remodelling and stiffening of resistance arteries in this model of diabetes mellitus.

Most studies using mineralocorticoid receptor antagonism in aldosterone-infused rats [3-5,7,9] or in stroke prone spontaneously hypertensive rats were performed after salt loading [18,19]. It has been suggested that aldosterone suppression may be impaired in salt loaded ZDF both with regard to plasma aldosterone concentrations [20] and tissue specific aldosterone synthase activity [21,22]. The detailed role of aldosterone in the salt induced aggravation of end organ damage in ZDF rats is unclear.

Aim of the present study was to test the impact of the specific mineralocorticoid antagonist eplerenone on salt-induced vascular remodeling and vascular stiffness in ZDF rats receiving normal and high dietary salt intake.

## Methods

### Animal experiments

The study was approved by the local committee on animal research and adhered for the "Guide for the Care and Use of Laboratory Animals" published by the US National Institutes of Health. Male ZDF (fa/fa) (Charles River Laboratories, Wilmington, MA) rats with a genetically induced form of type 2 diabetes including severe hyperglycemia and their normoglycemic controls, the Zucker lean (ZL) (Charles River Laboratories, Wilmington, MA) rats were studied. Rats were fed Purina 5008 rat chow (Charles River Laboratories) containing 29.2% protein, 7% fat, 52.3% carbohydrates, 4.3% fiber and 7.2% ash. They received tap water ad libitum. Rats were individually housed on a 12-h dark/12-h light cycle. After development of diabetes animals were divided into 7 groups. ZL and ZDF rats received either normal-salt (0.28% NaCl) (ZL, n = 9) (ZDF, n = 9) or high-salt diet (5.5% NaCl in chow) (ZL+S, n = 9) (ZDF+S, n = 8) for 10 weeks starting at an age of 15 weeks. Two additional groups of normal-salt and high-salt treated ZDF rats received eplerenone (100 mg/kg body weight per day in chow; Pharmacia/Pfizer) (ZDF + E, n = 8) (ZDF+S+E, n = 8). To control for effects of blood pressure lowering one group of ZDF rats on high salt diet received orally the vasodilator hydralazine (25 mg/kg body weight per day) (ZDF+S+H, n = 7). Serum glucose was measured weekly from an incision in the tail vein (Accu-Check Sensor, Roche, Mannheim, Germany). Systolic blood pressure (SBP) was measured weekly by the tail cuff method using an automated cuff inflator-pulse system (W+W electronic AG, BPrecorder No. 8005, Basel, Switzerland). The experiment was terminated at week 25 by decapitation of the rats. Blood was collected and plasma samples immediately frozen for further analysis. The mesenteric vasculature was dissected, and one segment was used for preparation of small arteries.

### Preparation and Study of Small Arteries

A 3rd order branch of the superior mesenteric artery was carefully dissected and mounted on a pressurized myograph (111 P; Danish Myo Technology, Aarhus, Denmark). Perfusion was performed using oxygenated 37°C Krebs solution (95% O_2_, 5% CO_2_) pH 7.4 containing (mmol^-1^): NaCl (118), KH_2_PO_4 _(1.18), KCl (4.7), MgSO_4 _(1.18), CaCl_2 _(2.5), D-Glucose (5.5), NAHCO_3 _(25) and EDTA (0.026) as described previously [23]. Vessels were equilibrated under a constant pressure of 45 mmHg. Vascular reactivity was tested with a single dose of norepinephrine (10^-5 ^mol/L^-1^). After precontraction with 10^-5 ^mol/L norepinephrine, endothelium dependent relaxation was assessed with acetylcholine (10^-4 ^mol/L). Vascular morphology and mechanics was assessed under resting conditions at three different points along the vessel. For assessment of passive mechanical properties, arteries were deactivated of myogenic tone by calcium-depletion (Ca^2+^-free KREBS solution containing EDTA 10 mmol/l for 30 min). Then intravascular pressure was increased from 3 to 10, 20, 30, and 40 mmHg and afterward in 20-mmHg steps up to 140 mmHg. The pressure was maintained at each pressure step until stable conditions were reached to allow the vessel to reach a steady-state diameter. The changes in internal diameter as well as media thickness of vessels in response to each increase in intravascular pressure were measured at three points along the vessel with the use of a calibrated video system (111 P, Danish Myo Technology, Aarhus, Denmark). At last vessels were fixed for histological analysis.

### Histology

Third order mesenteric arteries were pressurized at 45 mmHg, fixed with 2% paraformaldehyde solution at room temperature for 30 minutes, removed from the cannula, and processed for histological analysis. Paraffin embedded tissue sections of mesenteric arteries were stained with Sirius red F3BA (0,5% in saturated aqueous picric acid, Sigma Aldrich chemical Company, Steinheim, Germany). Collagen content in the media was quantified light microscopically with the Northern Eclipse imaging program (EM-PIX Imaging Inc.) and was determined by measuring the relative density per area in each Sirius red-stained section.

### Immunhistochemistry

Paraffin sections were dewaxed in sequential xylol and isopropyl alcohol to distilled water. Endogenous peroxidase activity was quenched by incubation with 3% H_2_O_2 _for 20 min at room temperature. For collagen IV examination sections were incubated with the primary antibody (goat anti-rat polyclonal antibody, 1:100, 37°C for 60 min; Southern biotech) after blocking with normal rabbit serum (30 min, room temperature). After rinsing in PBS, sections were incubated with the second antibody (biotinylated anti-goat, 1:100, 30 min, room temperature; Vector Laboratories, Inc., Burlingame, CA, USA). Antibody-antigen complexes were detected using a Vectastain Elite ABC kit (Vector). Then the label complex was applied (AEC Kit; Vector) for 7.5 min. Colour development was stopped under microscopic control by adding water, and sections were finally counterstained using haemoxylin. For examination of elastin content immunohistochemistry was performed in a similar manner. After blocking with normal goat serum (30 min, room temperature) rabbit anti-rat polyclonal antibody served as primary antibody (1:100 dilution, 37°C for 60 min; Chemicon International, Inc., Temecula, CA, USA). After rinsing in PBS, we used biotinylated anti-rabbit (1:100, 30 min, room temperature; Vector Laboratories, Inc., Burlingame, CA, USA) as second antibody and performed the further investigation steps identical to the collagen IV examination.

### Plasma Aldosterone Levels

Blood samples were centrifugated at 4°C, and the plasma was saved for subsequent analysis. Plasma aldosterone levels were measured by radioimmunoassay with the use of commercially available kit (DiaSorin, Dietzenbach, Germany), according to the manufacturer's instructions.

### Data Analysis

Data are presented as mean ± SEM. For systolic blood pressure levels, mean values of weekly measurements throughout active treatment were calculated for each animal. Circumferential stress which corresponds to wall tension or the distending force on the vessel wall was calculated as σ = (PD_i_)/(2 M), where P was intraluminal pressure, and D_i _and M were lumen diameter and media thickness, respectively. Circumferential strain which corresponds to pressure-induced relative increases in lumen diameter was calculated as ε = (D - D_0_)/D_0 _where D was the observed lumen diameter at a given intraluminal pressure and D_0 _was the original diameter measured at 3 mmHg, respectively. The strain-stress relation was fitted to an exponential curve for each vessel and the slope for each curve was determined. Groups were compared using 1-way ANOVA, or 2-way ANOVA for repeated measurements as appropriate. Post-hoc testing was performed using LSD test (1-way ANOVA and 2-way ANOVA for repeated measures). A p-value of < 0.05 was considered significant. Statistical analyses were performed using SigmaStat 3.0 software and SPSS 18 software (SPSS GmbH, Muenchen, Germany).

## Results

### Animal data

The non-diabetic ZL rats tended to start with a statistically insignificant lower body weight compared to the ZDF rats. At the end of the study there were no relevant differences in body weights (Table 1). ZL rats were normoglycemic irrespective of their salt intake. ZDF rats displayed significantly elevated serum glucose levels throughout the study. Interestingly, serum glucose levels were significantly lower in ZDF + S rats as compared with ZDF rats on the normal diet (Table 1).

**Table 1:** Biometric, serum and metabolic data:

	ZL	ZL+S	ZDF	ZDF+E	ZDF+S	ZDF+S+E	ZDF+S+H	P-value
**n**	9	9	9	8	8	8	7	
**Body weight at week 14 (g)**	325 ± 5	335 ± 7	341 ± 13	345 ± 9	357 ± 10	347 ± 8	360 ± 9	0.11
**Body weight at week 24 (g)**	381 ± 7	372 ± 8	362 ± 13	365 ± 13	371 ± 18	348 ± 10	369 ± 13	0.60
**Serum glucose levels (mg per 100 ml) at week 14**	95 ± 7*	96 ± 9*	377 ± 22	363 ± 15	344 ± 19	375 ± 7	389 ± 24	< 0.001
**Serum glucose levels (mg per 100 ml) at week 24**	91 ± 7*	83 ± 5*	408 ± 21	425 ± 27	319 ± 30♠	367 ± 34	364 ± 20	< 0.001
**Mean systolic blood pressure over treatment periode (mmHg)**	147 ± 3	150 ± 3	134 ± 3¥	138 ± 3	144 ± 4¥¥	139 ± 4	140 ± 3	< 0.001

Abbreviations: ZL: Zucker lean on normal diet. ZL + S: Zucker lean on high salt diet. ZDF: Zucker diabetic fatty. ZDF + E: Zucker diabetic fatty on eplerenone therapy. ZDF + S: Zucker diabetic fatty on high salt diet. ZDF + S + E: Zucker diabetic fatty on high salt diet with eplerenone therapy. ZDF + S + H: Zucker diabetic fatty on high salt diet with hydralazine therapy.

Post hoc testing: * P < 0.001 ZL and ZL + S vs. all other groups. ¥ P < 0.001 ZDF vs. ZL. ¥¥ P < 0.01 ZDF + S vs. ZDF. ♠ P < 0.05 ZDF + S vs. ZDF.

As previously reported [24], SBP was lower in ZDF rats compared to ZL rats at the start of the treatment. After 10 weeks of salt loading blood pressure was significantly higher in ZDF rats on high salt diet as compared to normal salt diet, while there was no significant difference between ZL rats on high and normal salt diet (Table 1). Both, eplerenone and hydralazine treatment did result in a not significant blood pressure reduction in salt loaded ZDF animals (- 5 mmHg ZDF+S+E, - 4 mmHg ZDF+S+H; p = ns) (Table 1). SBP was not different in the ZDF+E and ZDF+S+E groups. Thus, eplerenone prevented at least partially the salt induced rise in blood pressure.

**Figure 1:**
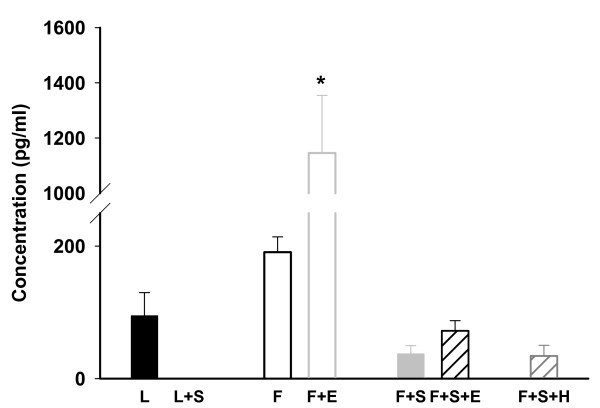
**Plasma aldosterone concentration (pg/ml)**. Analysis of variance * p < 0.001 ZDF + E vs. all other groups. L: Zucker lean on normal diet. L + S: Zucker lean on high salt diet. F: Zucker diabetic fatty. F + E: Zucker diabetic fatty on eplerenone therapy. F + S: Zucker diabetic fatty on high salt diet. F + S + E: Zucker diabetic fatty on high salt diet with eplerenone therapy. F + S + H: Zucker diabetic fatty on high salt diet with hydralazine therapy.

Confirming previous findings [17] plasma aldosterone levels were higher in ZDF than in ZL rats. High salt diet completely suppressed circulating aldosterone activity in normoglycemic ZL but not in diabetic ZDF rats (Figure 1).

### Morphology and Mechanical Properties of Mesenteric Arteries

Media thickness of mesenteric resistance arteries significantly increased with salt-loading in ZDF rats as previously reported [24]. Eplerenone and hydralazine prevented the increase of media thickness in animals on high-salt diet (Table 2). Media-to-lumen ratio was increased with higher salt intake (Table 2). This was prevented by eplerenone. Treatment with eplerenone was only effective in animals on high-salt diet. Hydralazine prevented the increase of media-to-lumen ratio to a similar extent like eplerenone in salt treated ZDF rats (Table 2).

**Table 2:** Structural, histological and immunohistochemical characteristics of mesenteric resistance arteries:

	ZL	ZL+S	ZDF	ZDF+E	ZDF+S	ZDF+S+E	ZDF+S+H	P-value
**Media (μm)**	17.9 ± 0.9	22.2 ± 1.9	19.8 ± 0.9	21.1 ± 1.1	28.7 ± 1.8◆	22.6 ± 2.0	23.6 ± 1.2	< 0.001
**Lumen (μm)**	297 ± 14	306 ± 22	298 ± 11	301 ± 21	273 ± 12	305 ± 13	302 ± 17	0.82
**Media/Lumen (%)**	6.1 ± 0.3	7.3 ± 0.3	6.7 ± 0.3	7.1 ± 0.3	10.7 ± 0.8♠	7.5 ± 0.7	7.9 ± 0.4	< 0.001
**Exponent** **Strain/stress relation**	6.4 ± 0.4	6.3 ± 0.3	6.5 ± 0.5	5.7 ± 0.3	7.5 ± 0.6	5.6 ± 0.3	6.4 ± 0.7	0.09
**Collagen IV (%)**	17.1 ± 0.7	25.9 ± 0.4*	20.9 ± 0.7§	18.3 ± 0.4¥¥	27.0 ± 0.4¥	21.3 ± 0.4¡	21.5 ± 0.5¡ ¡	< 0.001
**Elastin (%)**	21.6 ± 1.3	15.6 ± 1.2	18.8 ± 1.2	23.5 ± 1.4	17.6 ± 2.0	18.0 ± 1.2	19.2 ± 0.6	0.01
**Sirius red (%)**	12.1 ± 0.4	19.0 ± 0.9	16.2 ± 0.6	17.2 ± 0.6	19.6 ± 0.6♤	17.5 ± 0.9	17.1 ± 0.7✚	< 0.001

Abbreviations: ZL: Zucker lean on normal diet. ZL + S: Zucker lean on high salt diet. ZDF: Zucker diabetic fatty. ZDF + E: Zucker diabetic fatty on eplerenone therapy. ZDF + S: Zucker diabetic fatty on high salt diet. ZDF + S + E: Zucker diabetic fatty on high salt diet with eplerenone therapy. ZDF + S + H: Zucker diabetic fatty on high salt diet with hydralazine therapy.

Post hoc testing: * P < 0.001 ZL vs. ZL+S. § P < 0.001 ZL vs. ZDF. ¥ P < 0.001 ZDF vs. ZDF+S. ¥¥ p < 0.001 ZDF vs. ZDF+E. ¡ P < 0.001 ZDF+S vs. ZDF+S+E. ¡ ¡ p < 0.001 ZDF+S vs. ZDF+S+H. ◆ P < 0.05 ZDF+S vs. all other groups. ♠ P < 0.001 ZDF+S vs. all other groups. ♤ P < 0.05 ZDF vs. ZDF + S. ✚ p < 0.05 ZDF + S vs. ZDF + S + H.

**Figure 2:**
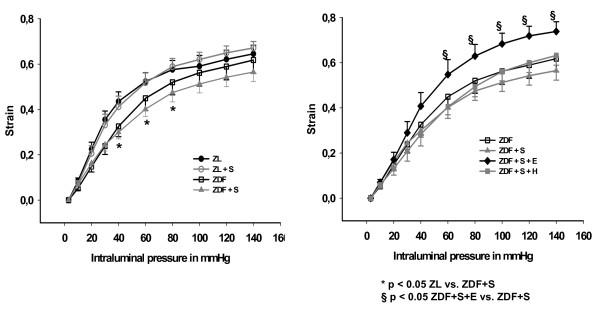
**Mechanical characteristics of mesenteric resistance arteries: Strain plotted against intraluminal pressure**. ZL: Zucker lean on normal diet. ZL + S: Zucker lean on high salt diet. ZDF: Zucker diabetic fatty. ZDF + S: Zucker diabetic fatty on high salt diet. ZDF + S + E: Zucker diabetic fatty on high salt diet with eplerenone therapy. ZDF + S + H: Zucker diabetic fatty on high salt diet with hydralazine therapy.

**Figure 3:**
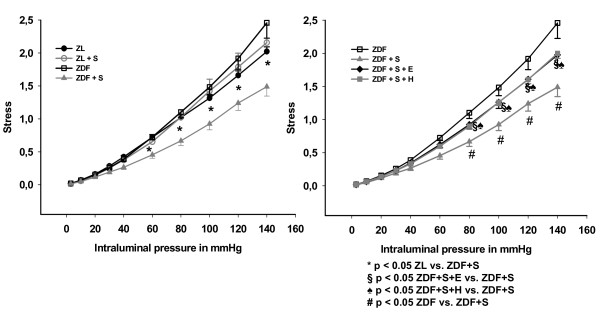
**Mechanical characteristics of mesenteric resistance arteries: Stress plotted against intraluminal pressure**. ZL: Zucker lean on normal diet. ZL + S: Zucker lean on high salt diet. ZDF: Zucker diabetic fatty. ZDF + S: Zucker diabetic fatty on high salt diet. ZDF + S + E: Zucker diabetic fatty on high salt diet with eplerenone therapy. ZDF + S + H: Zucker diabetic fatty on high salt diet with hydralazine therapy.

**Figure 4:**
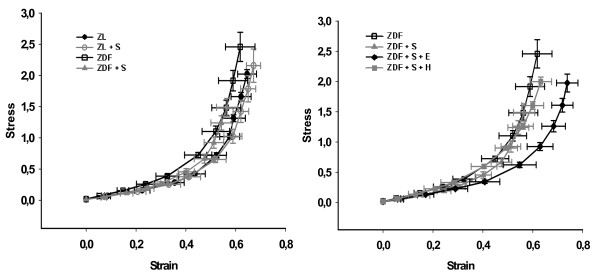
**Mechanical characteristics of mesenteric resistance arteries: Strain - Stress relationship**. ZL: Zucker lean on normal diet. ZL + S: Zucker lean on high salt diet. ZDF: Zucker diabetic fatty. ZDF + S: Zucker diabetic fatty on high salt diet. ZDF + S + E: Zucker diabetic fatty on high salt diet with eplerenone therapy. ZDF + S + H: Zucker diabetic fatty on high salt diet with hydralazine therapy.

The strain curve of arteries of salt-loaded ZDF rats was significantly lower when compared with ZL rats (p < 0.05) and when compared with salt-loaded ZDF rats treated with eplerenone (p < 0.05), but was not different compared to salt-loaded ZDF rats treated with hydralazine (p = 0.80) (Figure 2). This indicates increased vascular stiffness in salt-loaded ZDF rats, which could be prevented by eplerenone but not by hydralazine. The stress curve of arteries of salt-loaded ZDF rats was significantly lower when compared with ZL rats (p < 0.05), ZDF rats (p < 0.05), salt loaded ZDF rats treated with eplerenone (p < 0.05), and salt-loaded ZDF rats treated with hydralazine (p < 0.05), indicating that increased stiffness in salt loaded ZDF rats is at least in part geometry dependent (Figure 3). For further analysis of geometry independent stiffness the strain-stress curves were plotted. Eplerenone, but not hydralazine shifted the strain-stress curve to the right indicating a vascular wall composition with less resistant components (Figure 4). Accordingly, the exponent of the fitted exponential curve (Table 2) was increased in salt-treated ZDF rats. This increase was prevented by eplerenone but not by hydralazine. However, the significance niveau was missed (p = 0.09), which is likely due to the complex mathematical transformations necessary for this analysis.

### Collagen and Elastin Content of Mesenteric Arteries

**Figure 5:**
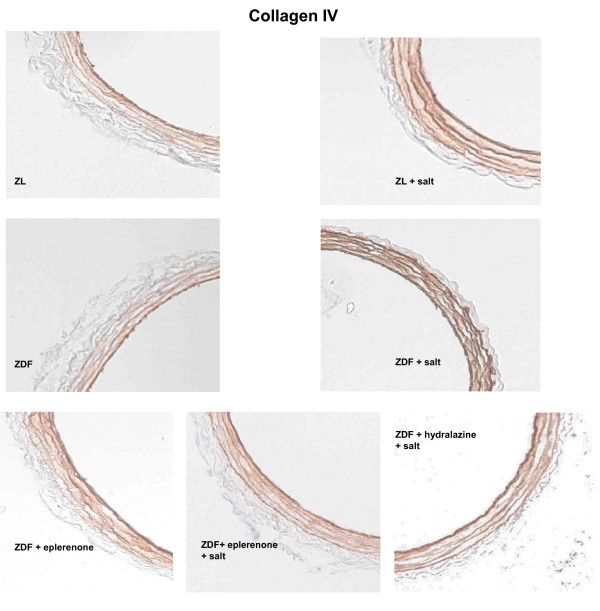
**Representative images of immunohistochemical staining of Collagen IV in the media of mesenteric resistance arteries**.

Collagen content in the media of mesenteric resistance arteries was significantly increased with salt loading (p < 0.001 ZL vs. ZL+S and p < 0.001 ZDF vs. ZDF+S for collagen IV). Eplerenone but also hydralazine treatment significantly reduced the content of media collagen IV in salt treated ZDF rats (p < 0.001; Table 2; Figure 5). Sirius red results paralleled the collagen IV findings indicating higher levels in salt treated groups that could be prevented both either with eplerenone (just missing the significance niveau with p = 0.062) or with hydralazine (p < 0.05). There were no significant differences in elastin content between the different treatment groups.

## Discussion

The major finding of our present study was, that specific inhibition of the mineralocorticoid aldosterone by eplerenone can effectively prevent stiffening of mesenteric resistance arteries in salt-loaded diabetic ZDF rats. The beneficial effect of eplerenone treatment on vascular stiffness was not present in the hydralazine treated control animals, indicating a specific impact of the aldosterone antagonism and not a non-specific effect of blood pressure reduction. Interestingly, we could observe that both agents resulted in ZDF+S animals in a similar reduction of the media-to-lumen-ratio as the usual measure to describe general vascular remodelling. This is likely to be mediated by the equal therapeutic efficacy on blood pressure reduction of eplerenone and hydralazine in our model. But eplerenone displayed an additional beneficial and apparently vessel geometry-independent effect on the development of vascular stiffness.

Indeed, analysis of the strain-stress curve for geometry-independent stiffness demonstrated decreased values in the eplerenone- but not in the hydralazine-treated ZDF+S rats. Analysis of the slopes of the strain-stress curves just missed the significance niveau, which is likely due to the complex mathematical transformations necessary for this analysis. Also other studies have found increased vascular stiffness in ZDF rats [25]. In that study the treatment with rosiglitazone was able to improve endothelial dysfunction but did not alter mechanical properties. However, it is to mention that this study was performed in a different vascular bed and size (femoral artery).

We performed histological analyses on cross sections of the mesenteric resistance arteries to gain insight into potential mechanisms responsible for the observed functional difference. The content of collagen IV was normalized in both ZDF+S+E and ZDF+S+H vessels to the levels of the ZDF animals with normal salt intake. Additionally, there was no effect of treatment in the ZDF+S rats with respect to the elastin content within the vessel wall.

At this time we can only speculate on possible other mechanisms, like fibronectin-content [9] or the arrangement of elastin [26]. Eplerenone reduced carotid artery stiffness in aldosterone-infused salt-loaded rats [9,11] in association with differences in fibronectin, but not in collagen or elastin density. Studies on human [27] and rat [28] cardiac myofibroblasts support a direct and blood pressure-independent effect of aldosterone on cardiac fibrosis. Previous studies demonstrated a beneficial effect of spironolactone on vascular hypertrophic remodelling and vascular function of mesenteric arteries in aldosterone-infused rats [13,14].

Some studies also show the effect of different levels of salt intake on aldosterone-induced end organ damage in hypertension. Most studies investigating the effect of aldosterone are performed in a salt-loaded state [3-5,7,9,18,19]. In a previous study in SHRSP rats we found an effect of eplerenone on resistance arteries only in salt-loaded animals [29]. High-salt induced cardiac hypertrophy and fibrosis in normotensive Wistar rats [30] and media hypertrophy in salt-fed normotensive Sprague-Dawley rats [31] could be prevented by spironolactone. Interestingly, both cardiac ventricles are affected by diabetes. A recent study showed decreased left and right ventricular function in ZDF rats [32]. However, the remodelling of the ventricles was found to be different with hypertrophy of the cardiomyocytes in the left ventricle and dilatation of the right ventricle without hypertrophy. An enhancement of local cardiac aldosterone production has been reported both in SHRSP compared with WKY rats [22] and under salt treatment in SHRSP [21] and WKY rats [33]. Therefore aldosterone associated end-organ damage may be attributed to direct effects of aldosterone on cardiovascular tissues. Moreover, we reported salt-sensitive blood-pressure in this ZDF model, which may be attributable to inadequate high levels of aldosterone and altered expression of renal SGK-1 dependent sodium transporter under high-salt treatment [24]. Under normal-salt conditions aldosterone levels are higher with less end-organ damage and less effect of aldosterone antagonism. This supports the degree to which high salt sensitizes tissues to aldosterone-induced cardiovascular injury.

A recent publication showed relevant gender differences in ZDF rats in development of diabetes, which is likely due to gender differences of hepatic anabolic pathways [34]. Another recent study has stressed the importance of impaired suppression of hepatic glucose production in ZDF rats [35]. Thus, we have to acknowledge that our findings may only be attributable to male ZDF rats.

## Conclusion

Findings from the present study indicate a pivotal role of the level of salt intake on the effect of aldosterone on vascular changes in diabetes mellitus. Our data provide important insights into the pathophysiological significance of salt in the role of mineralocorticoids such as aldosterone, and the therapeutic potential for mineralocorticoid receptor blockade for protection of the vasculature in diabetes mellitus. In summary our data suggest that consequent mineralocorticoid receptor antagonism might be an adjuvant therapy in preventing vascular complications of diabetes mellitus.

## Conflicts of interests

The authors declare that they have no competing interests.

## Authors' contributions

MR, SF, JW, and DE designed research; MR, PS, KA, CS, CB, DG, PK, SB and DE performed research; AL and GR revised the manuscript critically for intellectual content; MR and DE analyzed data; MR and DE wrote the paper. All authors have read and approved submission of the final manuscript.
